# Somatic mutation of DNAH genes implicated higher chemotherapy response rate in gastric adenocarcinoma patients

**DOI:** 10.1186/s12967-019-1867-6

**Published:** 2019-04-03

**Authors:** Chunchao Zhu, Qin Yang, Jia Xu, Wenyi Zhao, Zizhen Zhang, Danhua Xu, Yeqian Zhang, Enhao Zhao, Gang Zhao

**Affiliations:** 1 Department of Gastrointestinal Surgery, Ren Ji Hospital, School of Medicine, Shanghai Jiao Tong University, 160 Pujian Road, Shanghai, 200127 People’s Republic of China; 2 State Key Laboratory of Oncogenes and Related Genes, Shanghai Cancer Institute, Ren Ji Hospital, School of Medicine, Shanghai Jiao Tong University, Shanghai, People’s Republic of China

**Keywords:** Gastric cancer, DNAH mutation, TCGA, Chemotherapy response

## Abstract

**Background:**

The dynein axonemal heavy chain (DNAH) family of genes encode the dynein axonemal heavy chain, which is involved in cell motility. Genomic variations of DNAH family members have been frequently reported in diverse kinds of malignant tumors. In this study, we analyzed the genomic database to evaluate the mutation status of DNAH genes in gastric adenocarcinoma and further identified the significance of mutant DNAH genes as effective molecular biomarkers for predicting chemotherapy response in gastric cancer patients.

**Methods:**

We analyzed the clinical and genomic data of gastric cancer patients published in The Cancer Genome Atlas (TCGA) project. Data on chemotherapy response, overall survival (OS) and chemotherapy-free survival were retrieved. Then, we verified the results via targeted sequencing of gastric cancer patients with similar clinical characteristics but different chemotherapeutic outcomes.

**Results:**

In total, 132 gastric adenocarcinoma patients undergoing chemotherapy treatment from TCGA were included in our study. Somatic mutations in all 13 members of the DNAH family of genes were associated with different chemotherapy responses. Compared with patients with wild-type DNAH genes (n = 59), a significantly higher proportion of those with mutations in DNAH genes (n = 73) (55.9% vs 80.8%) responded to chemotherapy (P = 0.002). Moreover, DNAH mutations were correlated with significantly better OS (P = 0.027), chemotherapy-free survival (P = 0.027), fluoropyrimidine-free survival (P = 0.048) and platinum-free survival (P = 0.014). DNAH mutation status was an independent risk factor for OS (P = 0.015), chemotherapy-free survival (P = 0.015) and platinum-free survival (P = 0.011). We identified somatic mutations in 27 (42.2%) of the 64 stage III gastric adenocarcinoma patients receiving fluoropyrimidine-based chemotherapy by targeted exon sequencing with strict screening conditions. In our own cohort, a significantly higher proportion of patients (n = 32) with DNAH mutations than patients with wild-type DNAH genes (n = 32) had a good prognosis (OS > 48 months) (70.4% vs 35.1%) (P = 0.005).

**Conclusions:**

Dynein axonemal heavy chain gene mutations contribute positively to chemotherapy sensitivity in gastric cancer patients.

**Electronic supplementary material:**

The online version of this article (10.1186/s12967-019-1867-6) contains supplementary material, which is available to authorized users.

## Introduction

Gastric cancer remains one of the leading causes of death from malignant tumors worldwide [1]. As a type of highly heterogenetic malignant tumor [2], advanced or late stage gastric cancer is refractory to treatment. Despite some targeted therapeutic agents, such as herceptin [3, 4] and spatinib [5, 6], which have been used in the clinical setting, surgery and perioperative chemotherapy are still routine treatments for advanced gastric cancer [7, 8]. Unfortunately, chemotherapy resistance often occurs and causes treatment failure. The unpredictability of the chemotherapy response often makes the treatment regime ineffective and leads to the loss of optimal treatment opportunities.

Some molecular or genetic changes have been reported to predict the sensitivity to chemotherapeutics, such as thymidylate synthase or dihydropyrimidine dehydrogenase for fluoropyrimidine [9–11], *ERCC* mRNA levels and the *BMP4* epigenetic status for platinum-based chemotherapeutics [12, 13], and β-tubulin III for paclitaxel [14]. Additionally, BRCA2 mutations may contribute to the sensitivity to platinum-based chemotherapies in gastric cancer patients [15]. However, because of the lack of evidence, none of these potential biomarkers is routinely clinically used in gastric cancer treatment.

Because the response rate to single agent chemotherapy is poor, the majority of advanced gastric cancer patients receive combination regimes [16]. In spite of this, the overall efficacy of chemotherapy remains unsatisfying. Although it has been reported that there is a 76% response rate to the combined use of S1 (a combination of tegafur with two biomodulators, gimeracil and oteracil) and cisplatin, overall efficacy of the most commonly used regimes is less than 50% [17], which means that a large proportion of the patients did not obtain a survival benefit from chemotherapy but still experienced severe adverse drug reactions. Now that chemotherapy is an important treatment for modality for gastric cancer, a method of predicting the sensitivity to chemotherapy is needed. In this study, we aimed to identify the possible predictors of chemosensitivity by analyzing clinicopathologic data and exon mutation data from The Cancer Genome Atlas (TCGA). Then, further deep exon sequencing of cancer tissues was conducted to verify the results.

## Materials and methods

### Patients and study design

Because platinum and fluoropyrimidine are the most widely used chemotherapeutics in gastric cancer patients, we obtained whole-exon sequencing data and clinical information from TCGA for 132 gastric adenocarcinoma patients with who received that two chemotherapeutics postoperatively. Among these patients, 130 had complete follow-up information in the database. We performed two steps in our study. First, we analyzed the association between somatic mutations and chemotherapy sensitivity in TCGA cohort (n = 132). To analyze the data from TCGA, we considered “Complete Response”, “Partial Response” and “Stable Disease” to indicate chemosensitivity and considered “Clinical Progressive Disease” to indicate chemoresistance. Second, we validated the results from the first step through targeted exon sequencing of 64 pairs of cancerous and normal samples from prognostically selected gastric cancer patients who underwent surgery and chemotherapy. The 64 patients in our cohort were divided into two groups. Thirty-two patients had a good prognosis of overall survival (OS) (> 48 months after surgery), and 32 patients had a poor prognosis (< 12 months after surgery). All patients in our cohort had similar clinicopathologic features. Of the subjects, 50 (78.1%) were male, 14 (21.9%) were female, and the mean age was 60.8 years (range from 33 to 80 years old). All patients underwent radical gastrectomy and D2 lymph node resection without previous radiotherapy or chemotherapy from 2009 to 2011 and were identified as having stage IIIB and stage IIIC gastric cancer by postoperative pathologic diagnosis according to the American Joint Committee on Cancer (AJCC 7th Edition) staging system. These patients were treated with combination chemotherapy regimens involving fluoropyrimidine and/or platinum. Access to TCGA database was approved by the National Cancer Institute (https://tcga-data.nci.nih.gov/tcga). This project was approved by the ethics committee of Ren Ji Hospital, Shanghai Jiao Tong University School of Medicine with regard to the use of the samples. Informed consent was obtained from all patients before they were included in the study.

### Whole-exon sequencing data analysis

We analyzed the whole-exon sequencing data from the 132 TCGA cases with chemotherapy information. We calculated the mutation frequency in terms of the total number of nonsynonymous mutations, including single-nucleotide substitutions and insertion–deletion (indel) mutations. The proportions of different types of mutations including single-nucleotide variations (SNVs_ and indels in six possible mutation patterns (i.e., C > T, C > A, C > G, A > G, A > C and A > T) were calculated for every sample. Then, we analyzed the chemosensitivity rate based on the mutation status of every gene.

### Targeted-exon sequencing of DANH genes and data analysis

Formalin-fixed paraffin-embedded (FFPE) samples of gastric adenocarcinoma and paired normal gastric tissue samples were collected from individuals who underwent gastrectomy at the Ren Ji Hospital, Shanghai Jiao Tong University School of Medicine. We selected samples from gastric tumors that pathologists histologically identified as gastric adenocarcinoma according to the WHO classification system. DNA was extracted from the samples using the GenReadTM DNA FFPE Kit (Qiagen). The DNA quality was verified by the NanoDrop system and agarose gels, and at least 1 μg per sample was used for the construction of a library. The genomic DNA libraries were prepared using the protocols recommended by Illumina. The captured DNA libraries were sequenced with the Illumina HiSeq X Genome Analyzer, yielding 200 (2 × 100) base pairs in the final library fragments.

The sequencing reads were trimmed and filtered with Trimmomatic. The resulting reads were aligned to the hg19 reference genome using the Burrows–Wheeler Aligner (BWA), and the Genome Analysis Toolkit (GATK) was used for base quality score recalibration, indel realignment and duplicate removal. GATK 3.5, which contains the MuTect 2.0 module, was used to identify somatic SNVs in the whole-exome and targeted gene sequencing data. MuTect identifies candidate somatic SNVs by a Bayesian statistical analysis of the bases and their qualities in the tumor and normal BAM files at a given genomic locus. The somatic SNV and indel results were then combined and compared to the COSMIC database. Mutation functions were predicted using SnpEff, PolyPhen, PROVEAN and SIFT. A further filtering was performed by allele frequency; the filter program referenced previous studies [18] (Additional file 1: Table S1).

### Statistical analysis

The clinical and genomic data were analyzed using standard statistical tests, including χ^2^ tests and Fisher’s exact tests. Differences in survival were assessed using the log-rank test. All statistical tests were 2 sided, and P < 0.05 was considered statistically significant, with a confidence interval (CI) of 95%. The statistical analyses were performed with IBM SPSS version 20.0 and GraphPad Prism version 5.01.

## Results

### Association of DNAH genes mutations with the chemotherapy response in TCGA gastric cancer patients

In total, 132 TCGA gastric cancer cases were identified, including 58055 SNV and indel mutations in the exons. Of the 132 patients with recorded chemotherapy responses, 92 were classified as chemosensitive and 40 were classified as chemoresistant. The 58055 mutations were in 14833 genes, and *TP53* was the most frequently mutated gene. Interestingly, we found that DNAH (dynein axonemal heavy chain) genes were associated with the chemotherapy response rate. Initially, we found that the mutation rates of almost every member of the DNAH gene family were higher in chemosensitive patients than in chemoresistant patients. The mutation rates of all DNAH genes were as follows: *DNAH5* (22.8% vs 12.5%), *DNAH9* (22.8% vs 10.0%), *DNAH11* (17.4% vs 10.0%), *DNAH8* (18.5% vs 15.0%), *DNAH7* (16.3% vs 2.5%), *DNAH3* (14.1% vs 7.5%), *DNAH10* (14.1% vs 7.5%), *DNAH17* (13.0% vs 7.5%), *DNAH2* (12.0% vs 7.5%), *DNAH1* (8.7% vs 7.5%), *DNAH6* (3.3% vs 0), *DNAH12* (2.2% vs 0) and *DNAH14* (1.1% vs 0). We use the term “DNAH mutations” to refer to the mutations of these 13 members of the DNAH gene family, unless otherwise specified.

**Fig. 1:**
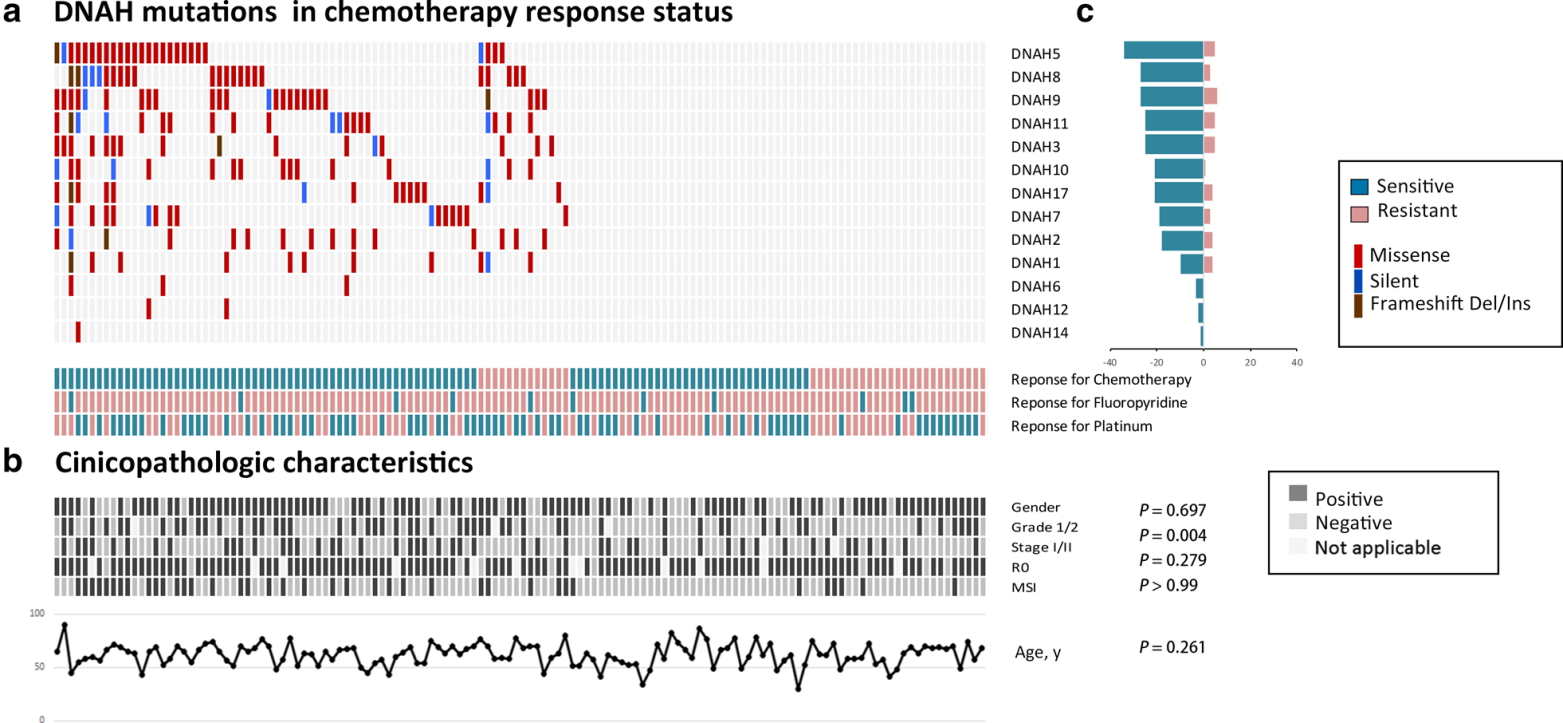
DNAH mutations that were detected in the 132 TCGA gastric cancer patients who underwent chemotherapy. **a** For each gene (row) indicated, tumors (columns) with mutations are labeled with red (missense mutations), blue (silent mutations), or brown (frameshift indels) bars. The lower labels with green (sensitive) bars and pink (resistant) bars represent the chemotherapy response status for each individual patient. **b** Clinicopathologic characteristics of each patient. “Gender” positive/negative indicates male/female; “Garde 1/2” positive/negative indicates that the level of tumor differentiation is well-moderate/poor according to the WHO classification system; “Stage I/II” positive/negative indicates that the tumor stage is I–II/III–IV according to the 7th AJCC cancer staging system; “R0” positive/negative denotes that the microscopic residual tumor was nonexistent/present after gastrectomy. The P value represents the result of the comparison between the patients with DNAH mutations vs those with wild-type DNAH genes; P < 0.05 indicates statistical significance. **c** Mutation counts for each gene are shown in **a**

To further analyze the DNAH genes, 73 of the 132 patients had DNAH gene mutations, and 59 of those patients (80.8%) were sensitive to chemotherapy (Fig. 1). However, in the other 59 patients without DNAH family gene mutations, the chemosensitivity rate was 55.9%. On the other hand, the proportions of mutated DNAH genes in the chemosensitive and chemoresistant groups were 64.1% and 35.0%, respectively; the difference was significant (P = 0.002, χ^2^ test). The analysis of the chemotherapeutics used in the 132 patients showed that DNAH mutations were significantly correlated with the response to fluoropyrimidine (P = 0.004, χ^2^ test) and platinum-based therapy (P = 0.005, Fisher’s exact test). In addition to the significant association with the response to chemotherapy, DNAH mutations were associated with tumor grade (P = 0.005, χ^2^ test) but not with clinical characteristics such as sex, age, TNM stage (AJCC 7th version), and R0 resection (Fig. 1).

### Association of DNAH mutations with TCGA gastric cancer patients survival

**Fig. 2:**
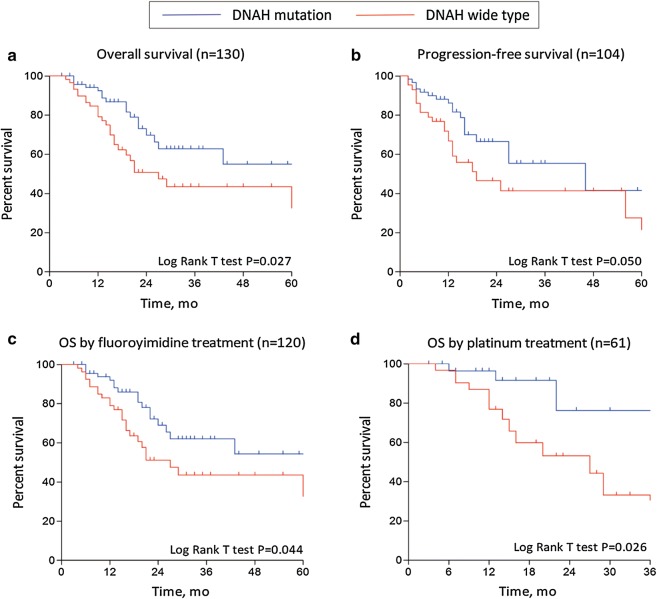
Estimates of the clinical outcome were made among patients who were stratified on the basis of DNAH mutations. Subgroups were compared with the log-rank test. The Kaplan–Meier analyses of overall survival (**a**), progression-free survival (**b**), fluoropyrimidine-free survival (**c**), and platinum-free survival of individuals (**d**) with gastric cancer in TCGA are shown

The χ^2^ test revealed that mutations in DNAH genes were significantly correlated with the chemotherapy response rate of gastric cancer patients, and then we identified the relationship between the prevalence of DNAH mutations and patient outcomes (Fig. 2). Because of missing follow-up data for 2 patients in TCGA, 130 of the 132 patients were included in the Kaplan–Meier survival analysis, which revealed that patients with DNAH mutations had a 3-year survival rate of approximately 62.8%, which was significantly longer than the survival rate of wild-type DNAH patients (P = 0.027, log-rank test). Additionally, the same trend was also found for progression-free survival (P = 0.050, log-rank test), although the difference was not significant.

Because DNAH mutations are associated with the rate of chemosensitivity to fluoropyrimidine and platinum, we generated the OS curves for gastric cancer patients receiving fluoropyrimidine and/or platinum treatment. The log-rank test revealed a significant difference in survival between gastric cancer patients with mutated and wild-type DNAH genes receiving fluoropyrimidine treatment (P = 0.044, log-rank test) and platinum-based treatment (P = 0.026, log-rank test).

### Association of DNAH genes mutations with chemotherapy-free survival rates

**Fig. 3:**
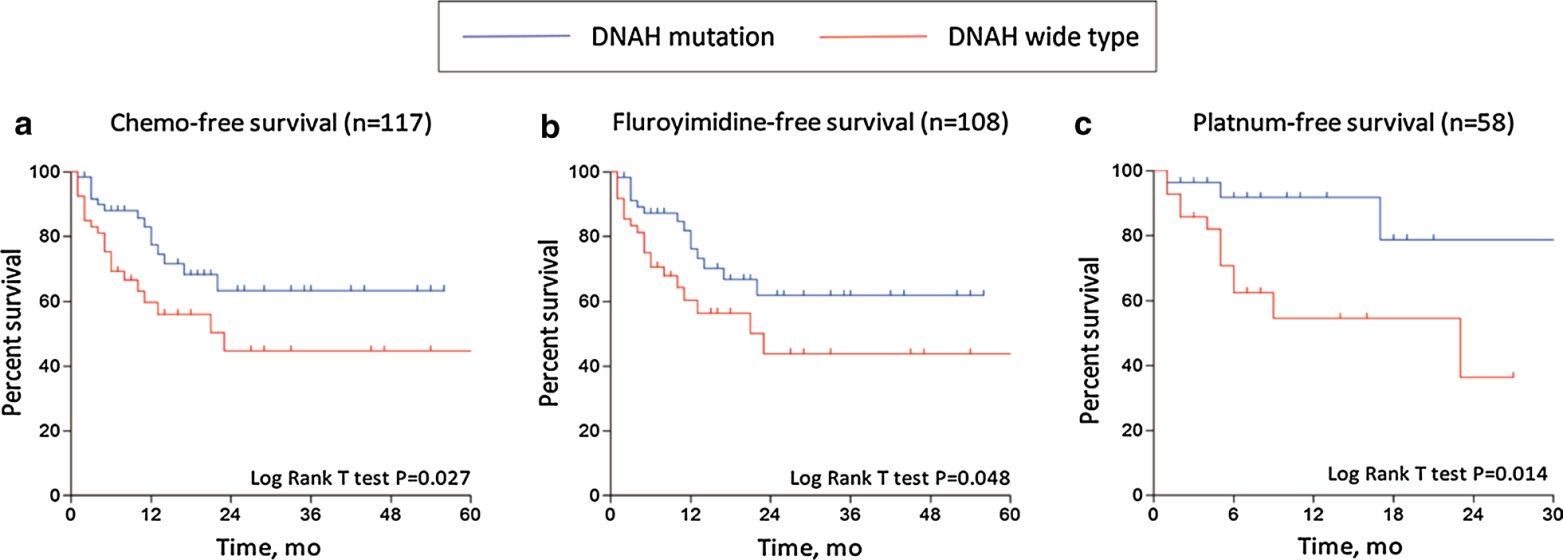
Clinical outcome with regard to chemotherapy-related survival. The subgroups were compared with the log-rank test. The Kaplan–Meier analyses of chemotherapy-free survival (**a**), fluoropyrimidine-free survival (**b**), and platinum-free survival (**c**) in individuals with gastric cancer in TCGA are shown

Sixty of the 73 patients with DNAH mutations who received postoperative chemotherapy in the TCGA were determined to be chemosensitive. Next, we analyzed the association between DNAH mutations and the chemotherapy-free period after treatment. In total, the chemotherapy-free survival rate was analyzed in 117 cases from TCGA. As shown in Fig. 3, the 3-year chemotherapy-free survival rate for patients with DNAH mutations was 63.3%, which was significantly higher than the rate for patients with wild-type DNAH genes (P = 0.027, log-rank test). For patients undergoing fluoropyrimidine and platinum-based chemotherapy, there were significant differences between patients with mutated and wild-type DNAH genes with regard to both fluoropyrimidine-free survival (P = 0.048, log-rank test) and platinum-free survival (P = 0.014, log-rank test). Univariate and multivariate Cox proportional hazards models showed that the DNAH mutation status is an independent risk factor for OS (P = 0.021) (Table 1) and is significantly associated with chemotherapy-free survival (P = 0.015); however, it is not a predictor of progression-free survival. Among the known prognostic factors, R0 resection is an independent risk factor (Table 2). Further analysis revealed that the effect of DNAH status on the chemotherapy-free duration may be mainly due to the administration of platinum-based treatment (P = 0.011).

**Table 1:** Univariate and multivariate models for overall survival and progression-free survival in 132 gastric cancer patients who underwent chemotherapy in The Cancer Genome Atlas (TCGA)

Characteristic	Univariate analysis		Multivariate analysis	
	HR (95% CI)	P value	HR (95% CI)	P value
Overall survival				
DNAH status	0.510 (0.276–0.0941)	*0.031*	0.457 (0.235–0.886)	*0.021*
TNM stage	1.841 (1.081–3.147)	*0.025*	1.367 (0.794–2.353)	0.259
R0 resection	4.699 (2.021–10.927)	<*0.001*	4.459 (1.689–11.773)	*0.003*
Grade	1.170 (0.636–2.152)	0.614		
Age at diagnosis, y	1.014 (0.985–1.044)	0.343		
Progression-free survival				
DNAH status	0.544 (0.291–1.016)	0.056		
TNM stage	0.952 (0.597–1.518)	0.873		
R0 resection	0.139 (0.746–8.157)	0.139		
Grade	2.303 (1.097–4.837)	*0.028*	2.303 (1.097–4.837)	*0.028*
Age at diagnosis, y	0.996 (0.966–1.026)	0.773		

Statistically significant values are in italics

**Table 2:** Univariate and multivariate models for chemotherapy-free survival in gastric cancer patients who underwent chemotherapy in The Cancer Genome Atlas (TCGA)

Characteristic	Univariate analysis		Multivariate analysis	
	HR (95% CI)	P value	HR (95% CI)	P value
Chemotherapy-free survival				
DNAH status	0.456 (0.248–0.848)	*0.011*	0.446 (0.233–0.856)	*0.015*
TNM stage	1.767 (1.010–3.092)	*0.046*	1.275 (0.716–2.268)	0.409
R0 resection	5.780 (2.464–13.558)	< *0.001*	5.289 (2.097–13.341)	< *0.001*
Grade	1.327 (0.722–2.439)	0.361		
Age at diagnosis, y	1.010 (0.982–1.308)	0.482		
Fluoropyrimidine-free survival				
DNAH status	0.518 (0.264–0.014)	0.055		
TNM stage	1.485 (1.189–5.192)	*0.016*	2.080 (0.961–4.502)	0.063
R0 resection	6.295 (2.338–16.963)	< *0.001*	5.136 (1.848–14.275)	*0.002*
Grade	1.429 (0.794–2.575)	0.234		
Age at diagnosis, y	1.019 (0.986–1.053)	0.269		
Platinum-free survival				
DNAH status	0.217 (0.061–0.768)	*0.018*	0.137 (0.030–0.629)	*0.011*
TNM stage	1.716 (0.761–3.872)	0.193		
R0 resection	4.184 (1.172–14.932)	*0.027*	5.116 (1.415–18.501)	*0.013*
Grade	1.415 (0.454–4.411)	0.550		
Age at diagnosis, y	1.000 (0.959–1.044)	0.988		

Statistically significant values are in italics

### Association of DNAH mutations with mutation spectra

**Fig. 4:**
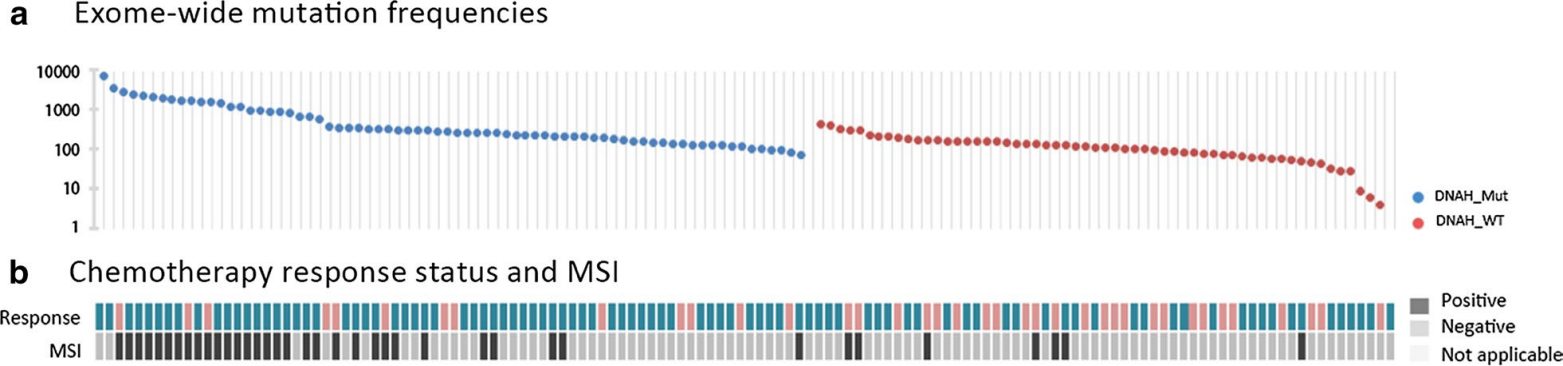
Association of DNAH mutations with mutation spectra in the TCGA cohort. **a** Exome-wide mutation frequencies in terms of the number of mutations (vertical axis) detected for each tumor (horizontal axis) in order of the decreasing number of mutations in each patient group stratified according to DNAH mutations. **b** Patients’ MS status and response to chemotherapy are also shown

**Fig. 5:**
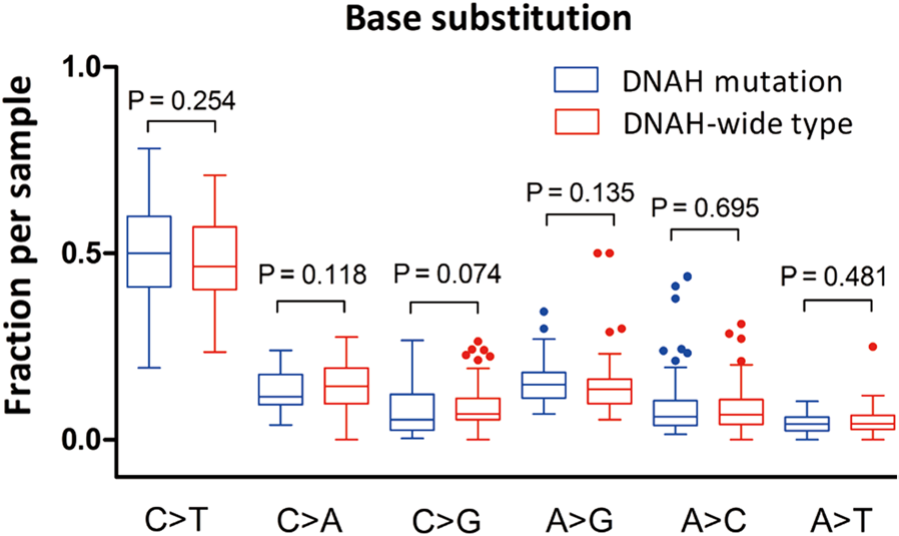
The base substitution analysis showed that DNAH mutations were not significantly associated with any of the six types of base transitions or transversions (C > T, C > A, C > G, A > G, A > C and A > T)

**Fig. 6:**
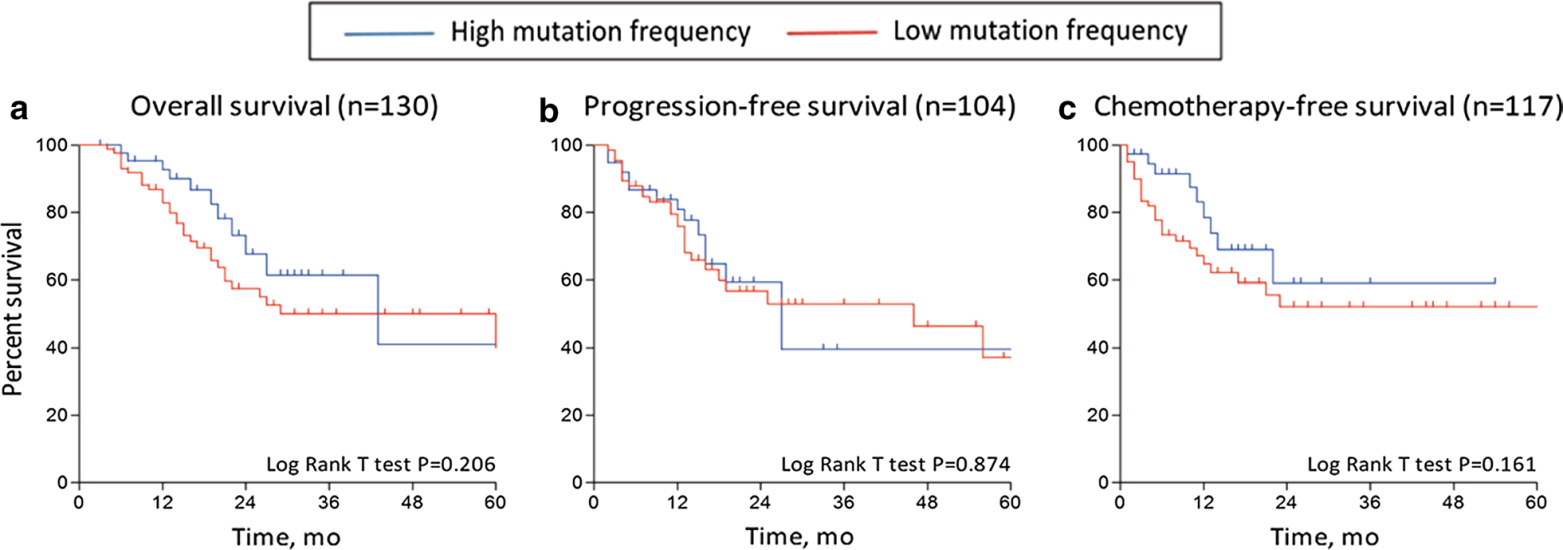
Kaplan–Meier analyses of overall survival, progression-free survival, and chemotherapy-free survival in patients stratified by mutation frequency. The patients with gastric cancer tumors were dichotomously categorized based on mutation rate into the following two groups: high (highest one-third, n = 44) and low (bottom two-thirds, n = 88) mutation frequency. Subgroups were compared via the log-rank test

Using the whole-exon sequencing data from 132 patients in TCGA, we performed a further analysis of the association between DNAH mutations and the mutation spectra in the exons of patients with gastric cancer. We found that the number of mutations in DNAH-mutated patients was significantly higher than the number in DNAH wild-type patients. The median number of mutations was 275 in patients with DNAH mutations vs 109 in patients with wild-type DNAH genes (P < 0.001, Mann–Whitney test). The microsatellite status was also associated with the DNAH mutation status (P < 0.001, χ^2^ test) (Fig. 4). The base substitution analysis showed that patients with DNAH mutations were not limited to any of the six types of base transitions or transversions (C > T, C > A, C > G, A > G, A > C and A > T) (Fig. 5). It was previously reported that a high mutation rate was correlated with a better prognosis in patients with endometrial [19], ovarian [20] and colorectal [21] cancer. Therefore, we analyzed the relationship between mutation rate and prognosis in the TCGA cohort in our study. We suppose the case with one-third of the topmost mutation number as the high mutation group and the remaining two-thirds as the low mutation group. However, the survival curve did not show a similar result to a previous report (Fig. 6). The result suggested that the difference in survival rate was not only determined by mutation frequency.

### Validation of the association between DNAH mutation and chemo-sensitive rate

**Fig. 7:**
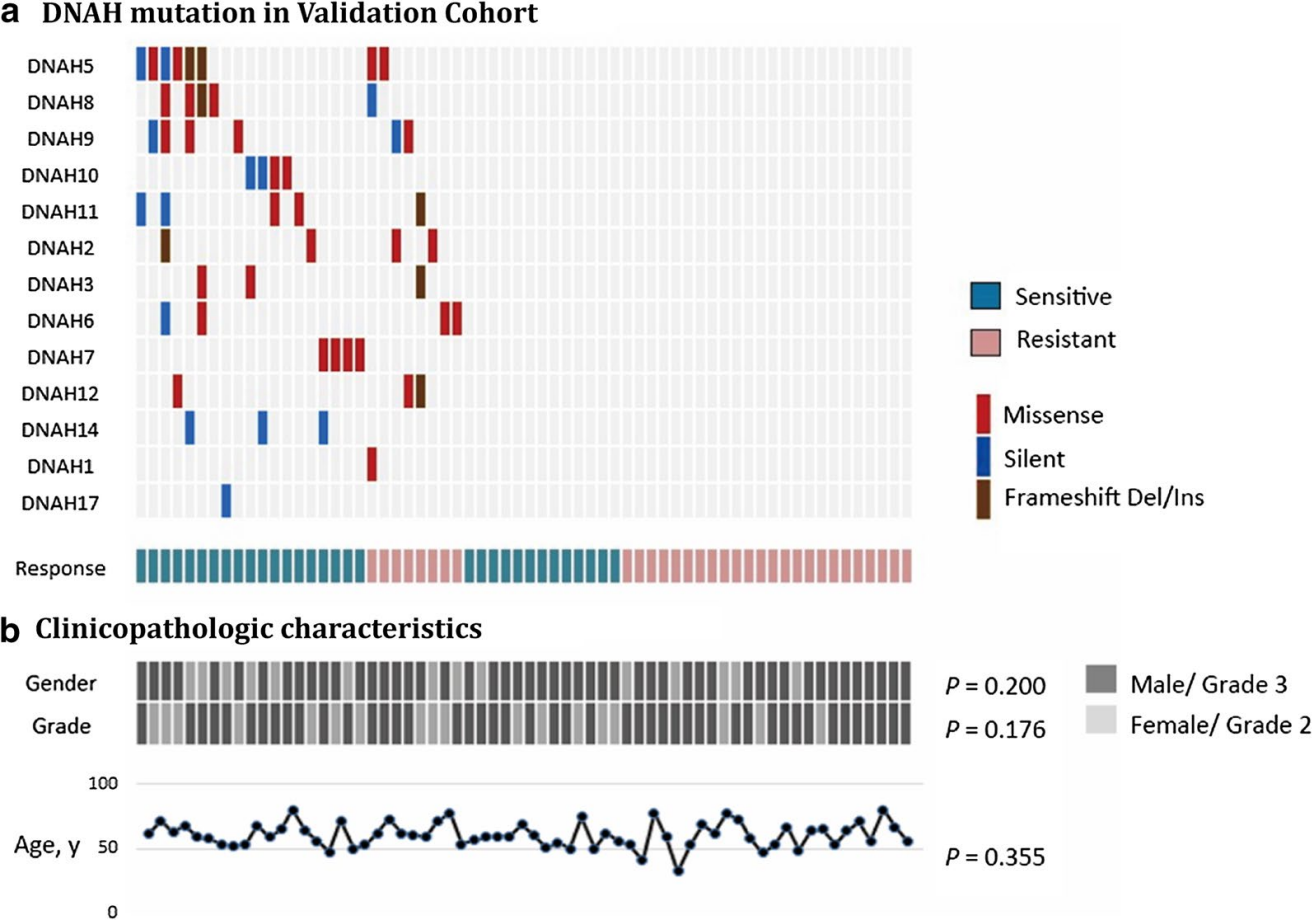
DNAH mutations detected by targeted sequencing in 64 gastric cancer patients who underwent chemotherapy. **a** For each gene (row) indicated, tumors (columns) with mutations are labeled with red (missense mutations), blue (silent mutations), or brown (frameshift indels) bars. The lower labels with green (sensitive) bars and pink (resistant) bars represent the chemotherapy response status of each individual patient. **b** Sex, Lauren classification and age are shown for each patient. The P value shows the result of the comparison between the patients with DNAH mutations vs patients with wild-type DNAH genes

To confirm the relationship between DNAH mutations and the response to chemotherapy, we examined the correlation from another point of view. We evaluated the DNAH mutation status in a prognostically selected and pathologically restricted population of patients with resected gastric cancer. We collected 64 FFPE samples from gastric adenocarcinoma patients who received radical gastrostomy and postoperative adjunctive chemotherapy and performed targeted sequencing of cancer-related genes, including the DNAH genes. Analysis of the data showed that DNAH mutation status, TNM stage and R0 resection are risk factors for OS, PFS or chemotherapy-free survival. To validate the association between mutations in the DNAH genes and the response to chemotherapy, we controlled for clinicopathological variables when selecting the patients. All 64 included patients had similar clinicopathologic characteristics (including TNM stage III cancer, poor differentiation, radical operation and postoperative chemotherapy) but substantially different prognoses. All patients received eight cycles of postoperative adjuvant chemotherapy with the combined use of platinum-based chemotherapy and fluoropyridines. Thirty-two patients who were determined to be chemoresistant died within 12 months of their operations. The other 32 patients who survived more than 48 months after their operations were determined to be chemosensitive. Twenty-seven patients in the cohort had mutations in the DNAH genes (Additional file 1: Table S2), and the distribution of mutations is shown in Fig. 7. The chemotherapy response rate was 70.4% for patients with DNAH mutation vs 35.1% for patients with wild-type DNAH genes (P = 0.005, χ^2^ test). With regard to the clinicopathological variables, the results showed that DNAH mutations were not related to tumor grade, sex or age.

## Discussion

Recurrence and metastasis after surgery is a major cause of treatment failure in patients with gastric cancer and contributes to the high mortality rate [22]. Although postoperative adjunct chemotherapy has been routinely used in advanced gastric cancer patients, tumor recurrence and progression are sometimes unavoidable [23]. Drug resistance is a major problem affecting treatment outcomes, and it has been the focus of recent research. The prediction of chemosensitivity in gastric cancer patients is important for the development of personalized treatment, with each patient treated according to his epigenetic and genetic background [24]. The ability to predict chemosensitivity is still lacking in clinical practice. Through the statistical analysis of data from TCGA, it has been reported that some novel gene variants are associated with chemotherapy response [20]; TCGA includes a large amount of data from different types of malignant tumors. In this study, we found that compared with wild-type DNAH genes, DNAH gene mutations in gastric cancer patients were significantly correlated with a higher chemotherapy response rate and better prognosis according to our analysis of data from TCGA. Moreover, we validated the results obtained from TCGA analysis through targeted sequencing of samples from our own cohort and we found that DNAH mutations were correlated with an improved response to chemotherapy. Thus, compared with gastric cancer patients with wild-type DNAH genes, those with DNAH mutations are more likely to benefit from chemotherapy. Of course, DNAH mutations cannot act as the sole predictors of chemosensitivity, and some patients with DNAH mutations had chemoresistant gastric cancer and poor outcomes.

Dynein axonemal heavy chain genes encode the axonemal dynein heavy chain protein, and alterations in DNAH genes have been initially detected in patients with primary ciliary dyskinesia [25, 26], sperm immobility [27] and some other diseases caused by cilia dysfunction. In recent years, alterations of DNAH genes have been frequently reported in several types of malignant tumors. For the first time, we identified an association between DNAH mutations and clinical outcomes in gastric cancer patients who were treated with chemotherapy. Structurally, dynein heavy chains are responsible for force production and ATPase activity, and they contain a highly conserved catalytic domain with 4 P-loop consensus motifs that is involved in nucleotide binding [28]. Two major classes of dynein, axonemal and cytoplasmic, have been identified. Axonemal dynein, found in cilia and flagella, are components of the outer or inner dynein arms attached to the microtubule doublets. There are no recurrent somatic mutation sites, as in BRCA2 and some other drug resistance-related genes.

Functionally, DNAH proteins affect ATPase activity [29], are involved in microtubule motor activity [30] and participate in several biological processes, such as cilium assembly, cilium movement and inner/outer dynein arm assembly [31]. In summary, axonemal heavy chain genes play important roles in ciliary assembly and cell motility. Thus, mutant DNAH genes may affect dynein motor complex functions, changing the microtubule-binding ability. Axonemal dyneins are involved in cilium- or flagellum-dependent motility, which might suggest a link between DNAH proteins and cell movement. Different types of cancer-related DNAH gene variants have been reported. DNAH2, DNAH5 and DNAH10 have been reported to have an elevated incidence of nonsynonymous single-nucleotide mutations and indels in CIMP-positive clear cell renal cell carcinomas [32]. An integrated analysis of RNA-Seq data and qRT-PCR results revealed that DNAH5 may play an important role in the development of colorectal cancer, and it might be useful for diagnosis, prognosis prediction and therapy [33]. DNAH8 has been reported to be a novel regulator of the androgen receptor that is associated with metastatic tumors and poor prognosis in patients with prostate cancer [34]. In invasive micropapillary carcinomas of the breast, DNAH9 has a substantially elevated mutation rate [35]. Considering the above evidence, DNAH genes may exert an effect on cancer development. As previously mentioned, the heavy chain protein of axonemal dynein that is encoded by the DNAH family of genes is an important component of microtubules. Microtubules have been reported as being important targets in cancer treatment. Mutations in DNAH genes influence the function of axonemal dynein and may further affect the structure of microtubules. Because the movement of tumor cells depends on this biological dynamic structure, we speculate that DNAH gene mutations may lead to the loss of function of these dynamic units; thus, DNAH gene mutations could be a protective factor, predisposing patients to a good prognosis and an improved chemotherapy response.

According to the results of previous studies, genetic instability could result in different responses to DNA-damaging agents [36]. A high mutation rate has been shown to be associated with a better prognosis in patients with malignant tumors, such as colorectal cancer and ovarian cancer. Nevertheless, we did not find a similar tendency in the 132 patients in TCGA who underwent chemotherapy. Platinum is a commonly used agent in chemotherapy for gastric cancer. The results from the analysis of the data from TCGA demonstrated that different chemotherapy responses may depend on the degree of sensitization to platinum, which was significantly different between DNAH-mutated and wild-type patients; the same result was not seen for fluoropyrimidine. On the basis of our results, although the DNAH gene mutation rate was correlated with genetic instability, the association with chemosensitivity was dependent on the chemotherapeutic agent.

We found that DNAH gene mutations are related to the response to chemotherapy in gastric cancer patients in this study; however, the mechanism underlying this correlation remains unclear. More studies are needed to clarify the underlying biological mechanism and evaluate the clinical values of DNAH gene mutations as predictors of chemosensitivity in gastric cancer patients.

## Conclusions

Using whole-exome sequencing data from TCGA, we identified DNAH genes as a novel group of chemosensitivity-related genes. The reliability of this result was verified by targeted sequencing of our own samples. Compared with patients with wild-type DNAH genes, gastric cancer patients with DNAH gene mutations were associated with longer survival times and higher chemotherapy sensitivity rates. This finding may indicate that DNAH mutations could be a clinical predictor of chemotherapy sensitivity. DNAH gene mutations may be an important addition to the existing chemotherapy response predictors.

## Additional file

**Additional file 1: Table S1.** The filtering conditions to detect somatic SNV. **Table S2.** DNAH mutations in our cohort with 64 FFPE samples of gastric adenocarcinoma.

## Abbreviations

TCGA: The Cancer Genome Atlas
OS: overall survival
FFPE: formalin-fixed-paraffin-embedded
BWA: Burrows–Wheeler Aligner
GATK: Genome Analysis Toolkit
DHCs: dynein heavy chains
DNAH: dynein axonemal heavy chain
CI: confidence interval
